# Calomel and “Eclecticism”

**Published:** 1852-01

**Authors:** 


*Calomel and "Eclecticism."*—We notice in an "eclectic" periodical entitled "The American Medical and Surgical Journal," published at Syracuse, N. Y., the following remarks on the use of calomel:—"The *Hydrargyri Chloridum Mite* has, for several centuries, been employed as a remedy for disease, with much utility and success. Candor requires of us this acknowledgement in justice to our old school brethren, and, perhaps, to some professed eclectics who dispense it to their patients."

The candor of this admission strikes us as somewhat remarkable. What, then, becomes of *eclecticism* "falsely so called?"

